# Validation and depth evaluation of recurrent neural network‐based ultra low‐pass genome sequencing for the detection of absence of heterozygosity: A multi‐centre study of 409 cases

**DOI:** 10.1002/ctm2.1752

**Published:** 2024-07-04

**Authors:** Yeqing Qian, Jianjun Zhu, Zhiguo Tang, Yan Sun, Zhonghua Wang, Fei Tang, Yun Yang, Linlin Fan, Yixi Sun, Bei Liu, Min Chen, Yuqin Luo, Junjie Hu, Kai Yan, Jianfen Man, Lina Wang, Cangcang Jia, Ping Tang, Xinyi Zhu, Chaohong Wang, Junxiang Tang, Yuanyuan Xia, Xueqin Guo, Kang Zhang, Xiaoli Wang, Suping Li, Lijie Song, Jiansheng Zhu, Minyue Dong

**Affiliations:** ^1^ Department of Reproductive Genetics, Women's Hospital, Zhejiang University School of Medicine Hangzhou China; ^2^ Key Laboratory of Reproductive Genetics, Ministry of Education, School of Medicine Zhejiang University Hangzhou China; ^3^ Department of Fetal Medical Center Jiaxing Maternity and Children Health Care Hospital, Jiaxing University Jiaxing China; ^4^ Andrological Medicine, Maternity and Child Health Hospital of Anhui Province Affiliated Maternity and Child Health Hospital of Anhui Medical University Hefei China; ^5^ BGI Genomics Shenzhen China; ^6^ Clin Lab, BGI Genomics Tianjin China; ^7^ Clin Lab, BGI Genomics Wuhan China; ^8^ Medical Genetics Center, Maternity and Child Health Hospital of Anhui Province Affiliated Maternity and Child Health Hospital of Anhui Medical University Hefei China; ^9^ Clin Lab, BGI Genomics Shenzhen China; ^10^ DTU Bioengineering, Technical University of Denmark 2800 Kongens Lyngby Denmark

Dear Editor,

We conducted a comprehensive clinical assessment of our newly developed method, CNVseq‐AOH, for the detection of absence of heterozygosity (AOH) using low‐pass genome sequencing (LP GS) with ultra‐low sequencing data in this multi‐centre study.

Although AOH in chromosomes does not necessarily have clinical consequences, the detection of AOH is clinically important when it is related to imprinting effects or autosomal recessive disease mechanisms.^1^, ^2^, ^3^, ^4^ LP GS (also known as CNVseq) has enabled the detection of copy‐number variants (CNVs) for its superiority in sensitivity and specificity.^2^, ^5^, ^6^ In China, it has been recommended as a first‐line diagnostic method for foetuses displaying structural abnormalities.^7^ However, the investigation on the use of LP GS for the detection of AOH is limited, and furthermore, LP GS has never been reported for the detection of AOH in a low‐pass setting of less than 1‐fold. To establish a comprehensive clinical assessment of our newly developed method (https://github.com/helplessness/CNVseq‐AOH), CNVseq‐AOH, for the detection of AOH, and to investigate the optimal sequencing depth, we gathered 409 samples (by far the largest clinical samples) of amniotic fluid from three hospitals. Our data showed that CNVseq‐AOH in large‐scale clinical practice maintained high sensitivity (100%) and specificity (100%), providing good evidence for its clinical application. This multi‐centre study demonstrated the feasibility of CNVseq‐AOH for the detection of AOH in real clinical settings.

First, using samples with positive AOH regions from the 1000 Genomes Project (1KGP), we performed concordant analysis for CNVseq‐AOH and chromosomal microarray analysis (CMA). CNVseq‐AOH was aimed to predict AOH using LP GS with ultra‐low sequencing data. Compared to CMA, CNVseq‐AOH showed high sensitivity and great potential to improve the genetic testing of AOH.

Second, to assess and comprehensively study the performance of CNVseq‐AOH in a real clinical environment, a total of 409 archived DNA samples of amniotic fluid (209 with positive AOH results (Table S1) and 200 samples with negative AOH results by CMA (Table S3)) from Women's Hospital, Zhejiang University School of Medicine, Jiaxing Maternity and Child Health Care Hospital and Anhui Province Maternity & Child Health Hospital from April 2017 to March 2023, were recruited. Among the 409 samples, CMA identified 209 cases with positive AOHs (506 AOH regions) (Figure 1). LP GS for the 409 samples were conducted on the MGISEQ‐2000 platform for single‐end (35 bp) sequencing as previously described.^8^ For these samples, an average of 71.48 M uniquely aligned high quality reads (UAHRs) were obtained, approximately 0.83‐fold for each sample. After sequencing, AOH detection was performed for each sample using CNVseq‐AOH.

**FIGURE 1:**
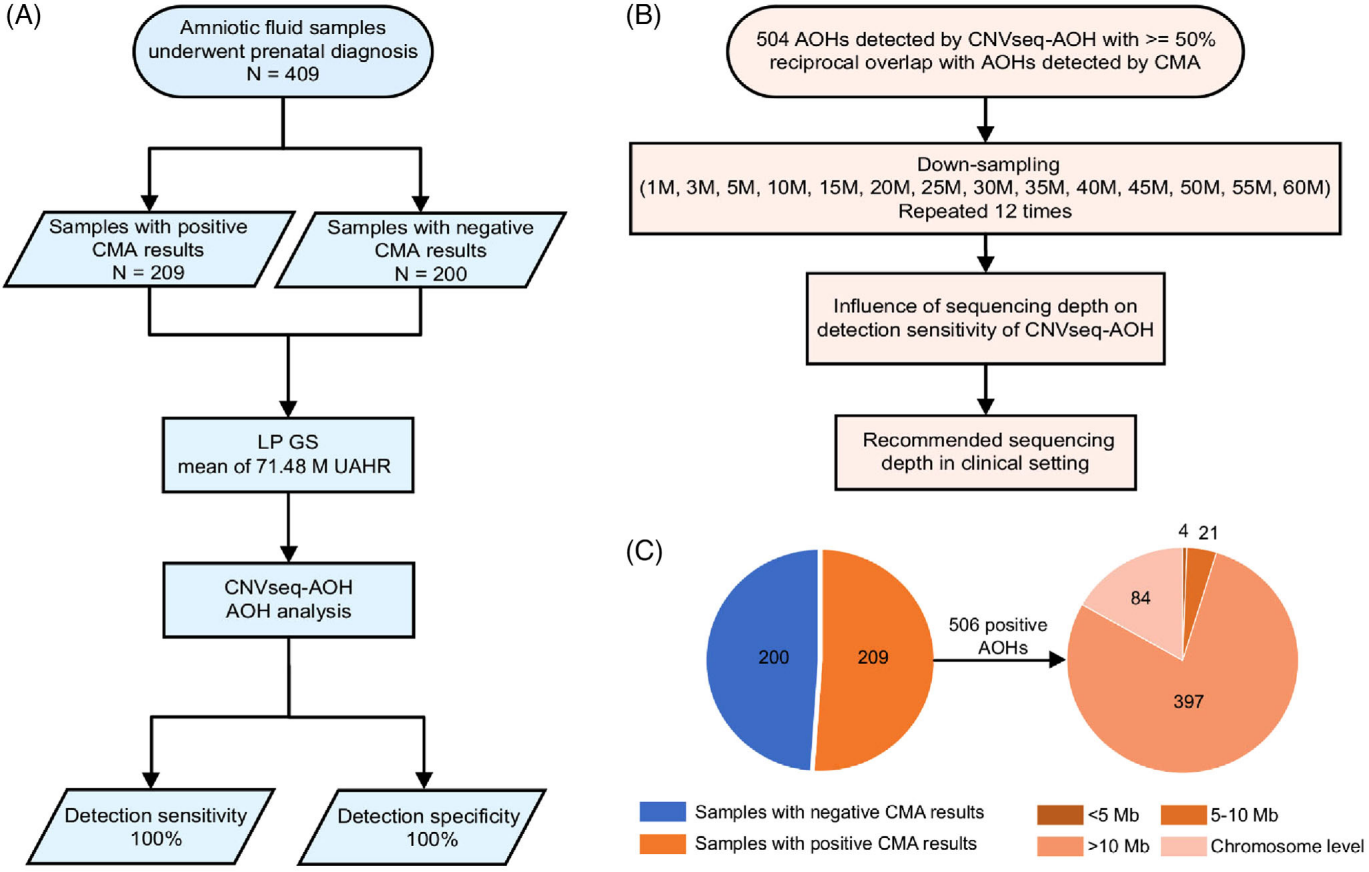
Study design. (A) Study workflow; (B) Depth evaluation workflow; (C) The pie chart of 506 absence of heterozygosity (AOH) regions detected in 209 samples with positive chromosomal microarray analysis (CMA) results, including four AOHs < 5 Mb, 21 AOHs with a size between 5 and 10 M, 397 AOHs > 10 Mb and 84 chromosomal level AOHs.

The results of CMA were blinded to individuals who were analysing using CNVseq‐AOH. When using the CMA results as a reference, the diagnostic yield of CNVseq‐AOH was found to be equivalent to that of CMA (Table S1). Specifically, CNVseq‐AOH demonstrated a sensitivity of 100% (209/209) and a specificity of 100% (200/200) in our cohort.

The overlap for the 506 AOH regions detected by CMA and CNVseq‐AOH was further analysed (Table S1). It showed that ∼99.60% (504/506) of the AOHs detected by CNVseq‐AOH had a reciprocal overlap of more than 50% with the AOHs detected by CMA (Table S2). Compared with CMA, two AOHs were detected to be with an overlap of less than 50% in case PS201 (Figure 2) and case PS44 (Figure 3). Case PS201 included 1 positive AOH across the whole chromosome 9 by CMA. In the CNVseq‐AOH detection results, the region was divided into multiple subregions (Figure 2C), with a total overlap of less than 50% between the two methods. Further analysis revealed low‐level mosaicism (∼6.7%) of the whole chromosome 9 (Figure 2E). The presence of this mosaicism can affect the performance of CNVseq‐AOH, leading to the discrepancy observed between the two methods. For the ∼10.4 Mb AOH in case PS44, abnormal signals were detected in the reported regions of CMA (Figure 3C), with some regions showing relatively dispersed signals from allele difference analysis (Figure 3F). These differences may be due to the differences in detection principles between the two methods.

**FIGURE 2:**
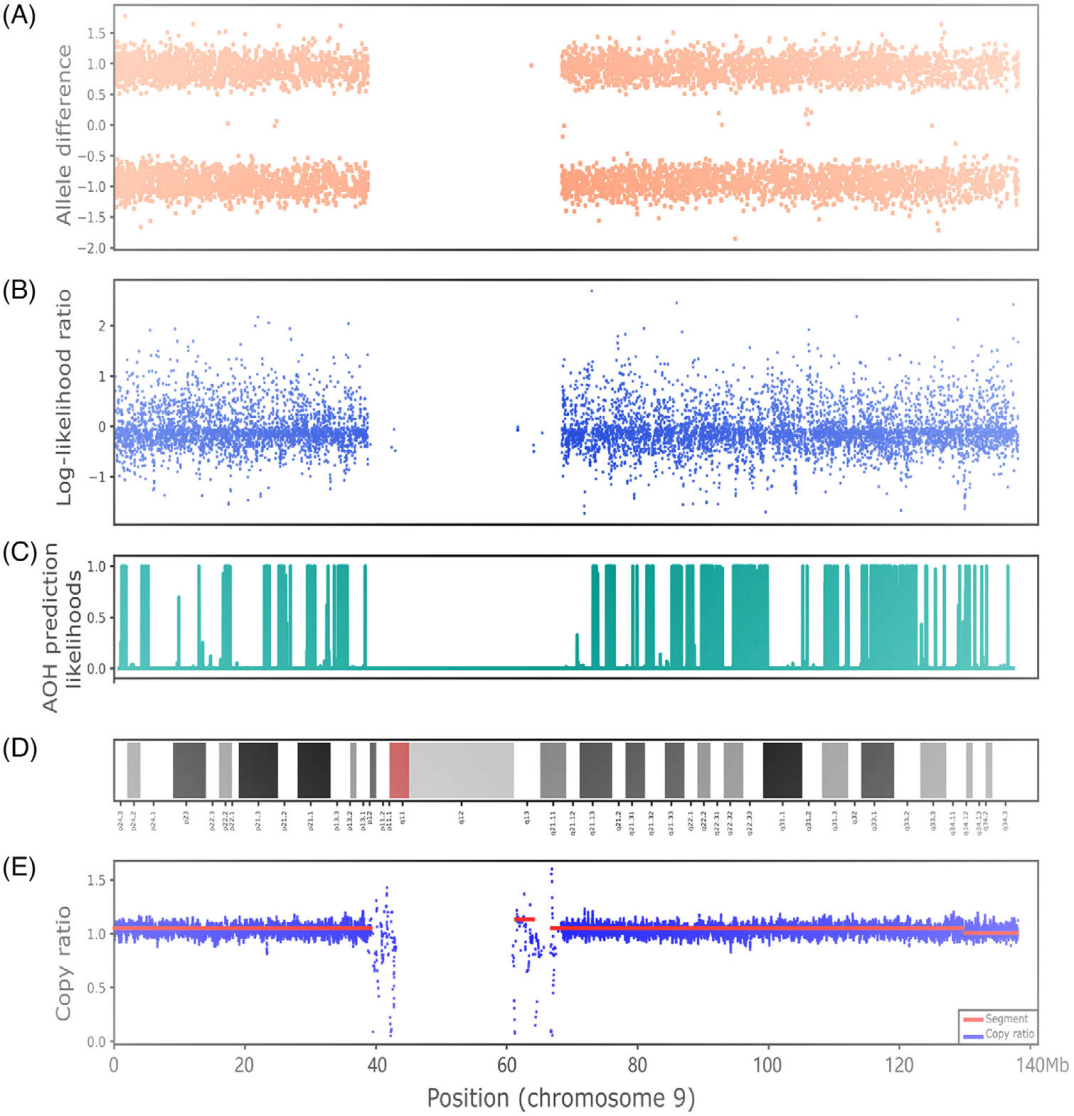
Whole chromosome 9 results for case PS201. (A) Chromosomal microarray analysis (CMA) results; (B) Log‐likelihood ratio for haploid and diploid in each bin; (C) Absence of heterozygosity (AOH) prediction likelihoods of CNVseq‐AOH; (D) Diagram of chromosome 9; (E) Copy ratio for chromosome 9.

**FIGURE 3:**
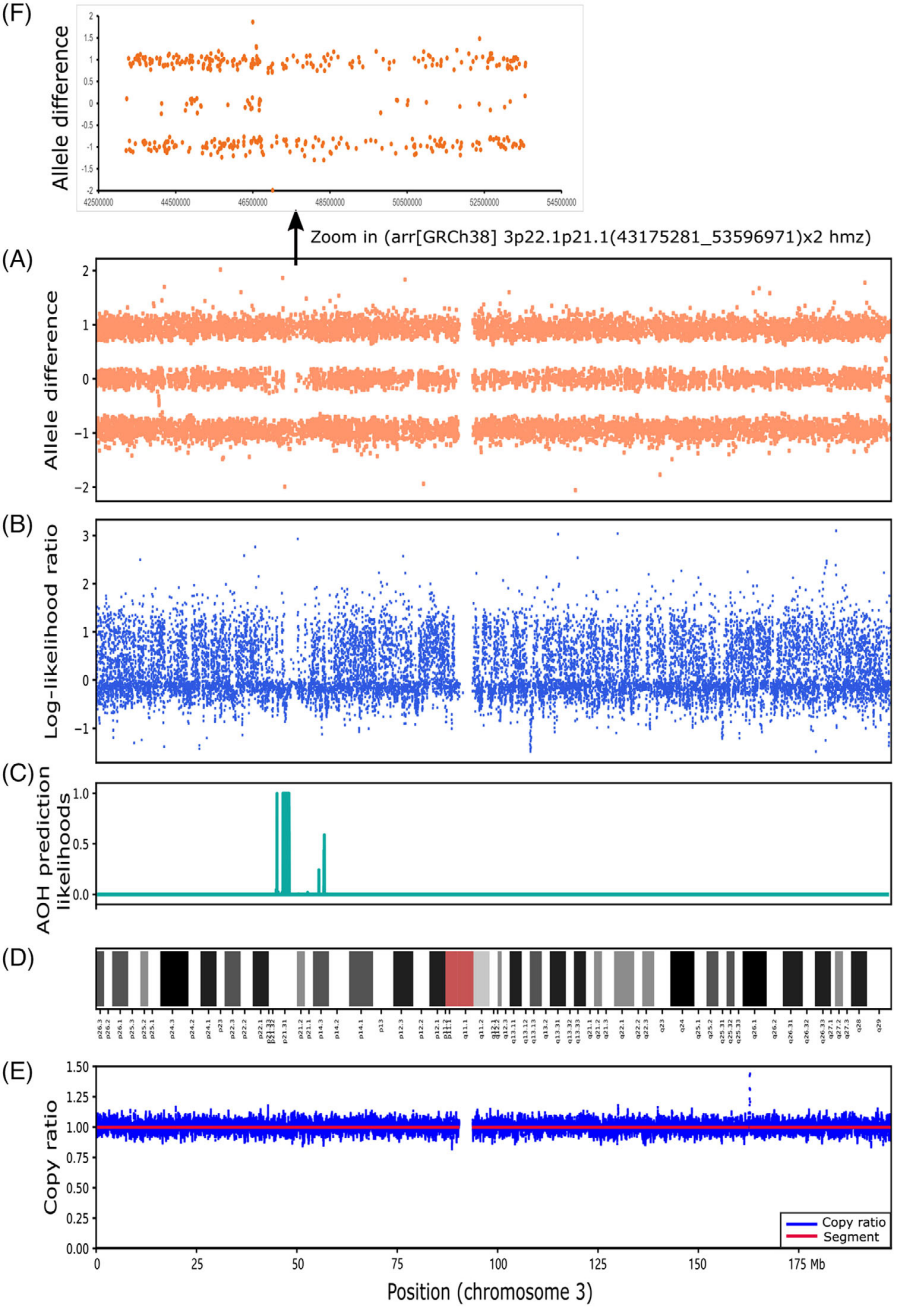
Chromosome 3 results for case PS44. (A) Chromosomal microarray analysis (CMA) results; (B) Log‐likelihood ratio for haploid and diploid in each bin; (C) Absence of heterozygosity (AOH) prediction likelihoods of CNVseq‐AOH; (D) Diagram of chromosome 3; (E) Copy ratio for chromosome 3; (F) Detailed allele difference in the reported region of arr[GRCh38]3p22.1p21.1(43175281 53596971)x2 hmz by CMA.

Third, to examine how the detection sensitivity of CNVseq‐AOH is affected by sequencing depth, depth evaluation was performed (Figure 1B). In total, the UAHRs for 504 AOHs were utilized to create downsampling samples (14 different sequencing depths for each sample). As a result, the performance of CNVseq‐AOH in downsampling samples varied depending on the size of the AOH (Figure 4 and Table S2). The detection sensitivity of 4 AOHs (cases PS1‐4) with sizes less than 5 Mb was greatly influenced by UAHR. The detection sensitivity of AOHs with sizes between 5 and 10 Mb was also influenced by UAHR (Figure 4) and reached a plateau at 20 M UAHR. For AOHs larger than 10 Mb and at the chromosome level, the impact of sequencing depth becomes less pronounced (Figure 4 and Table S2), reaching 99.74% and 99.89% at 10 M UAHR. Overall, the detection sensitivity tended to increase as the number of UAHRs increased, and it reached a plateau at 15 M UAHRs for all the 504 AOHs (Figure 4). When using 15 M UAHRs, the overall detection sensitivity is over 99.66%. Therefore, 15 M UAHRs were considered optimal for detecting AOHs using CNVseq‐AOH based on our cohort, approximately 23 times (342.86 M reads) less than the existing method.^9^

**FIGURE 4:**
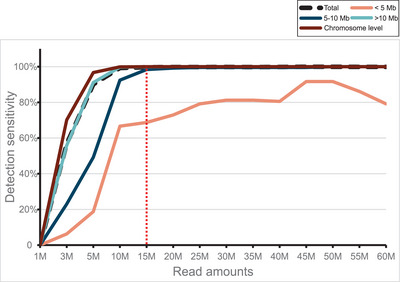
Depth evaluation. Detection sensitivity of CNVseq‐AOH for downsampling samples. The dotted red line shows the optimal uniquely aligned high quality reads (UAHR) (15 M).

What is more, CNVseq‐AOH addressed the long‐standing challenge of standard LP GS in detecting AOH. Typically, the cost of CMA for one sample is more than $600.^10^ In our laboratory, the overall cost of LP GS for a single case was approximately $248.^8^ Combining CNVseq‐AOH, LP GS could achieve the same level of performance as CMA for AOH detection (> 10 Mb) at a very low sequencing depth.

In summary, we developed a method for predicting AOH using LP GS data and tested its performance in this multi‐centre study. CNVseq‐AOH can identify regions of AOH accurately in the clinical setting and possesses great potential to improve the genetic testing of AOH. According to these findings, CNVseq‐AOH has demonstrated high precision in detecting AOHs, indicating its promising application in clinical settings.

## AUTHOR CONTRIBUTIONS

Suping Li, Lijie Song, Jiansheng Zhu, Minyue Dong, Yeqing Qian, Jianjun Zhu, Zhiguo Tang and Yan Sun contributed to the conception and design of the study. Yan Sun wrote the first draft of the article. Yun Yang, Linlin Fan, Yixi Sun, Bei Liu, Min Chen, Yuqin Luo, Junjie Hu and Kai Yan designed and performed the experiments. Yan Sun, Zhonghua Wang, Fei Tang, Jianfen Man, Lina Wang, Cangcang Jia, Ping Tang, Xinyi Zhu, Chaohong Wang, Junxiang Tang, Yuanyuan Xia, Xueqin Guo, Kang Zhang and Xiaoli Wang performed data analysis. Yeqing Qian, Lijie Song, Minyue Dong and Yan Sun contributed to revising the manuscript. All authors reviewed the manuscript and approved the submitted version.

## CONFLICT OF INTEREST STATEMENT

The authors declare no conflict of interest.

## FUNDING INFORMATION

This study was supported by the National Key R&D Program of China (2023YFC2705600). This work was also supported by the 4+X Clinical Research Project of Women's Hospital, Zhejiang University School of Medicine (ZDFY2023‐4XPY201), Technology Bureau of Jiaxing, Zhejiang Province (2023AY31030), Anhui Key Research and Development Program (2022e07020031), and Zhejiang Provincial Natural Science Foundation of China (LY22H110004). These projects are non‐profit research projects by the government and had no role in the study design, data collection and analysis, decision to publish, or preparation of the manuscript.

## ETHICS STATEMENT

This study was approved by the Institutional Review Board of Women's Hospital, Zhejiang University School of Medicine (NO. IRB‐20230313‐R), Jiaxing Maternity and Child Health Care Hospital (NO. 2023−047), Anhui Province Maternity & Child Health Hospital (NO. 2023‐005‐01) and the Institutional Review Board of BGI (NO. BGI‐IRB 23140).

## Supporting information

Supporting Information

Supporting Information

## Data Availability

The data generated and analysed during the current study is not publicly available as they are patient samples and sharing them could compromise research participant privacy. The data that support the findings of this study are available on request from the corresponding author.
