# Supplementary material for: Validation and depth evaluation of recurrent neural network‐based ultra low‐pass genome sequencing for the detection of absence of heterozygosity: A multi‐centre study of 409 cases
Source: Clin Transl Med. 2024 Jul 4;14(7):e1752. doi: 10.1002/ctm2.1752 (PMC11223989; doi:10.1002/ctm2.1752)
Supplement: Supplementary file 1 — Supporting Information [file CTM2-14-e1752-s001.docx]

**Validation and depth evaluation of RNN-based ultra low-pass genome sequencing for the detection of AOH: a multi-center study of 409 cases**

**Supplementary methods**

**LP GS and CNVseq-AOH**

In general, LP GS for the 409 samples were conducted as previously described on the MGISEQ-2000 for single-end (35bp) sequencing (BGISEQ-2000RS high-throughput sequencing kit, SE35, V3.0; MGI Tech Co) [1]. According to our previous report, 25 M uniquely aligned high quality reads are optimal for detecting most aneuploidies and microdeletions/microduplications [1].

For the detection of AOH, our preliminarily analysis concluded (based on the analysis of down-sampling samples from the 1KGP) that CNVseq-AOH can predict AOHs with a concordant rate of 99.66% with only 0.1-fold LP GS data for AOHs larger than 10 Mb. However, using 0.1-fold LP GS data, CNVseq-AOH can only generate a concordant rate of 59.49% for AOHs on the X chromosome (unpublished data). It seems that the detection sensitivity of CNVseq-AOH may be improved by improving the sequencing depth of LP GS. In this context, the specific amount of data required for clinical testing needs to be further investigated using real clinical samples. In our labs, 10 samples were loaded together and sequenced on 1 lane of BGISEQ-2000RS for prenatal diagnosis, resulting approximately 35 reads (~0.3-fold).

Taking into account our preliminarily results on 1KGP, we doubled the amount of routine data for the 409 samples, with 5 samples per lane. This could generate ~70 Mb reads theoretically, which is sufficient for the following depth evaluation analysis.

**References**

[1] Qian, Y., Sun, Y., Guo, X., Song, L., Sun, Y., Gao, X., Liu, B., Xu, Y., Chen, N., Chen, M., et al. (2023). Validation and depth evaluation of low-pass genome sequencing in prenatal diagnosis using 387 amniotic fluid samples. J Med Genet 60, 933-938.
